# Gut microbiota and colorectal cancer

**DOI:** 10.1186/s41021-016-0038-8

**Published:** 2016-06-01

**Authors:** Mayuko Yamamoto, Satoshi Matsumoto

**Affiliations:** Yakult Central Institute, 5-11 Izumi, Kunitachi-shi, Tokyo, 186-8650 Japan

**Keywords:** Gut microbiota, Colorectal cancer, Colitis-associated cancer, Mucosal immune system, Dysbiosis

## Abstract

The mucosal immune system is unique to the gastrointestinal mucosa, in which a large number of immune cells are located and exert multiple functions. Meanwhile, ~100 trillion microorganisms are thought to co-inhabit in the gastrointestinal tract. Furthermore, immune cells and gut microbiota have a mutual influence and the maintenance of this symbiotic relationship results in gut homeostasis. A recent study suggested that a disturbance of gut microbiota—so called “dysbiosis”—is related to various diseases, such as inflammatory bowel disease (IBD) and colitis-associated cancer (CAC). In this review, we discuss the relationship between gut microbiota and the mucosal immune system with regard to the development of IBD and CAC. In addition, we elucidate the possibility of probiotics in treatment against these diseases.

## Background

The mammalian gastrointestinal tract, where digestion and absorption occurs, acts as the frontline of defense against microorganisms from the environment. Therefore there is an established unique immune surveillance system called the mucosal immune system. It is supposed that a half of immunocompetent cells reside in the gut mucosa, and the balance between them with a variety of properties, including T helper 17 (Th17) cells and regulatory T (T_reg_) cells, is thought to be controlled exquisitely. Characteristics of the mucosal immune system are represented by their contrasted immune functions, such as the removal of pathogens and immune unresponsiveness to the food antigens and indigenous gut microbiota. Increasing evidence suggests that the gut microbiota also plays key roles in homeostatic maintenance of the mucosal immune system. Imbalance of gut microbiota, so called “dysbiosis”, based on dysregulation of the mucosal immune system affects the development and pathogenesis of various diseases such as allergy, diabetes, autoimmune diseases and cancer [1, 2]. In addition, a recent finding has suggested that when feces of healthy adults were intra-rectally inoculated into patients with recurrent *Clostridium difficile* infection, the symptoms improved in association with the recovery from dysbiosis [3], which would be a clear example of gut microbiota contributing to restraint of colonic inflammation. Furthermore regarding the onset of inflammatory bowel disease (IBD) and colitis-associated cancer (CAC), the interaction between the mucosal immune system and gut microbiota is important, because in germfree animal models of these diseases, no symptoms are observed [4, 5]. In this review, we discuss the roles of gut microbiota and the mucosal immune system on the development of IBD and CAC.

## Review

### Gut microbiota in IBD

IBD is categorized into Crohn’s disease (CD) and ulcerative colitis (UC) based on pathophysiological characteristics. UC is an inflammatory disease confined to the colonic mucosa, whereas CD has the potential to develop along the entire gastrointestinal tract with a higher occurrence in the small and large intestines. Because both diseases exhibit repeated remission and relapse, it is important that we urgently improve the quality of life of patients with IBD. In accordance with the development of an analytical method, based on bacterial 16S rDNA and next generation sequencing (NGS), the characteristics of gut microbiota in patients with IBD are rapidly being elucidated. A loss of bacterial diversity and dysbiosis is present in the gut microbiota of patients with IBD, as commonly detected using NGS. In particular, there is a marked decrease in the occupancy of *Firmicutes* and *Bacteroidetes* in gut microbiota, which normally predominates in a healthy adult. It has been reported that 46 strains of *Clostridium* derived from mice and 17 strains of *Clostridium* derived from humans induced differentiation of Foxp3^+^ T_reg_ cells, resulting in mass production of IL-10, via augmentation of TGF-β provided by colonic epithelial cells [6, 7]. It was then demonstrated that *Clostridium butyricum* when used as a probiotic, could induce IL-10 production from macrophages in colonic mucosa, which resulted in suppression of acute colitis in mice [8]. It has been discussed that butyrate participated in suppression of colitis and colorectal cancer. The bacterial metabolite, butyrate, induces the differentiation of colonic Foxp3^+^ T_reg_ cells and ameliorates the development of colitis. A possible mechanism for this regulation of differentiation may be that butyrate enhances histone H3 acetylation in the promoter and conserved, non-coding sequence regions of the Foxp3 locus [9]. Because the occupancy of *Clostridium* clusters IV and XIVa, in which numerous butyrate-producing bacteria exist, have been shown to be decreased in the gut microbiota of patients with IBD, it would be expected that clinical applications of these results would follow.

With regard to the interaction of the mucosal immune system and gut microbiota, secretory immunoglobulin A (IgA) is important. The presence of secretory IgA in the intestinal lumen is indispensable for the exclusion of pathogenic germs and neutralization of toxins. Germ-free mice have few IgA-producing cells in their intestinal mucosa. Total bacterial numbers increase markedly in the mice deleted activation induced cytidine deaminase (AID) gene, which is normally essential for somatic hypermutation and class switch recombination during IgA gene rearrangement. IgA produced in inhibitory receptor of immune system (programmed cell death-1 (PD-1)) gene-deficient mice had a low affinity for bacteria, which caused alterations of microbial communities in the gut [10]. In addition, it has been recently reported that some gut microbiota was coated with IgA, and IgA-coated fecal bacteria taken from patients with IBD exacerbated dextran sulfate sodium (DSS)-induced colitis in gnotobiotic mice [11].

### Gut microbiota in colorectal cancer

Colorectal cancer is one of the most common fatal malignancies in the world. The involvement of gut microbiota in the development of colorectal cancer has been noted for some time. IL-10-deficient mice and TCRβ/p53 double knockout mice do not develop colorectal cancer under germfree environment, providing a rationale for the association between colorectal cancer and gut microbiota [12]. Chronic inflammation is known to predispose an individual to cancer, and as such, the presence of IBD increases the risk of colorectal cancer. Another such example would be CAC. The molecular mechanisms underlying the pathogenesis of CAC are unclear and do not follow the adenoma-carcinoma sequence [13]. It is urgent to clarify the mechanism underlying development of CAC, because ~20 % of patients with chronic inflammation in the form of UC develop CAC within 30 years from the onset, with at least half of the cases resulting in death. A recent study demonstrated that dysbiosis of gut micobiota plays a key role in the pathophysiology of CAC. Bacterial diversity is remarkably decreased in gut microbiota of sporadic colorectal cancer and CAC mice models. When gnotobiotic mice are colonized with feces taken from sporadic colorectal cancer or CAC mice, the incidence and number of tumors are increased in both cases, compared with those colonized with feces of healthy mice. CAC can be experimentally induced in rodent models by the combination of introduction to azoxymethane (AOM) and repeated exposure to the inflammatory agent DSS. Results from time-course analysis of the composition of gut microbiota during development of CAC in this model indicated tumor-bearing mice showed enrichment in operational taxonomic units (OTUs) affiliated with members of the *Bacteroides*, *Odoribacter*, and *Allobaculum* genera and decreases in OTUs affiliated with members of the *Prevotellaceae* and *Porphyromonadaceae* families. Furthermore, conventionalization (colonization of germfree mice with gut microbiota) with tumor-bearing mice significantly increased colon tumorigenesis compared to those colonized with feces of healthy mice [14]. However, mice exposed to the chemical mutagen do not develop tumors if they receive antibiotics and mice that received feces of tumor-bearing mice do not develop tumors if they are not exposed to the mutagen. These findings suggest that gut microbiota plays a part in the initiation of colorectal cancer. CAC results from the complex relationship between chronic inflammation and dysbiosis of gut microbiota, which would induce irreversible changes to intestinal epithelial cells. *Bacteroides fragilis* toxin, produced by enterotoxigenic *B. fragilis* (ETBF), triggers colorectal cancer by binding to colonic epithelial cells and stimulating cleavage of the cell adhesion molecule E-cadherin, which act as the tumor suppressor protein [15]. Antibody-mediated blockade of interleukin-17 (IL-17), a key cytokine for proinflammatory responses, inhibits ETBF-induced colitis and tumor formation [16]. Gut microbiota of IL-10 deficient mice developing spontaneously severe colitis have decreases in bacterial diversity and increases in the occupancy of *Enterobacteriaceae* [17]. IL-10 deficient mice colonized with either *Escherichia coli* (*E. coli*) or *Enterococcus faecalis* develop colon inflammation, but only the mice receiving *E. coli* developed colon tumors. Moreover, it was reported that Colibactin, the product of polyketide synthase (pks) in *E. coli* NC101, cleaved double stranded DNA in colonic epithelial cells and promoted invasive carcinoma in AOM-treated IL-10deficient mice [18]. Because the expression of the ETBF toxin gene and pks gene of *E. coli* NC101 is higher in patients with colorectal cancer when compared to healthy adults, aberrant proliferation of these bacteria caused by dysbiosis of gut microbiota would induce disruption of epithelial barrier function and contribute to the mechanism of CAC development. However, there is some uncertainty, because the murine AOM/DSS model administered microbes from patients with colorectal cancer developed unexpectedly fewer tumors than those that received bacteria from healthy human donors [19]. Therefore, we would need to validate the evidence, accumulated by studies using animal models of colorectal cancer, in human. Furthermore, it has also been revealed that the role of gut microbiota in cancer extended to treatment, influencing not only the effectiveness of chemotherapy but also its side effects. Both germfree mice and antibiotic-treated mice show cyclophosphamide resistance and in these mice, pathogenic Th17 cells are shown to be decreased [20]. Taken together, it is likely that modulating the gut microbiota will become an effective tool to combat colorectal cancer.

### CAC and IL-6/Stat3 pathway

**Fig. 1:**
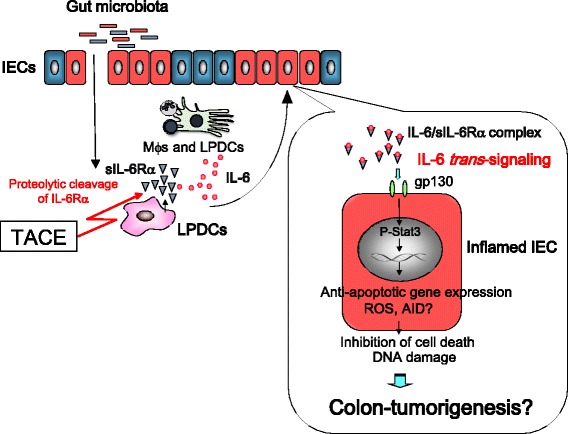
Representative scheme of how IL-6 *trans*-signaling modulates inflammation-based colorectal tumorigenesis. Under inflammatory conditions, sIL-6Rα is generated from LPDCs by TACE, which proteolytically cleaves the extracellular domain of membrane-bound IL-6Rα. Gut microbiota had a key role on the activation of TACE. IL-6 is also produced by macrophages (Mϕs) and DCs in LP and binds to sIL-6Rα. The IL-6/sIL-6Rα complex can associate with gp130 and induces IL-6 signal transduction through the phosphorylation of Stat3, termed IL-6 *trans*-signaling. IL-6 *trans*-signaling triggered in LP inputs its downstream signal into intestinal epithelial cells (IECs) and induces the expression of anti-apoptotic gene and AID and the production of reactive oxygen species (ROS), which leads to the inhibition of cell death, genetic instability and DNA damage. It is speculated that long-term accumulation of IL-6 *trans*-signaling finally leads to colon tumorigenesis

**Fig. 2:**
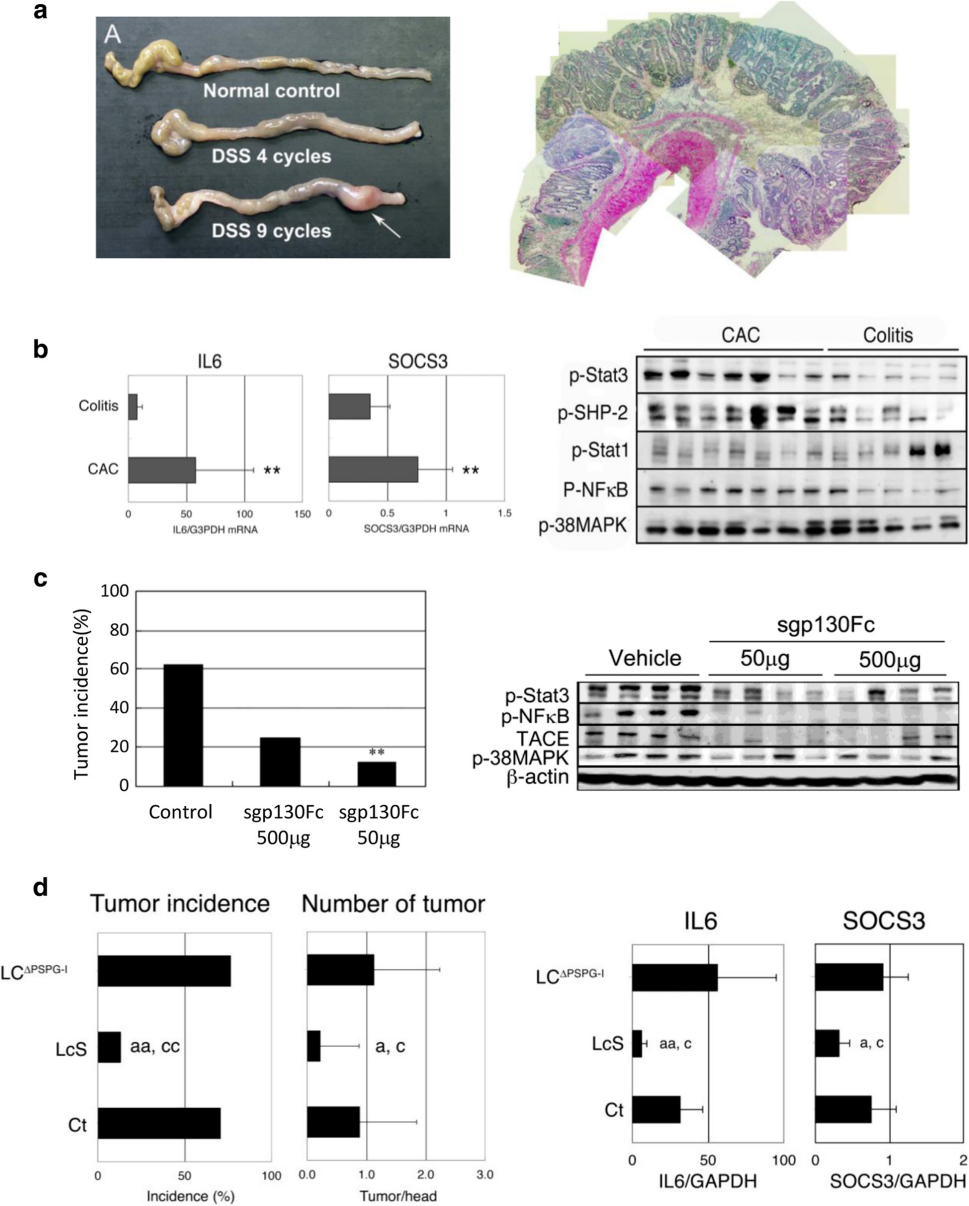
Characteristics of a murine model of CAC and the possibility of probiotic treatment in the prevention of CAC. A-*left*, Stereomicroscopic observation of a murine model of DSS-induced CAC. CAC was induced in BALB/c mice by nine cycles of treatment with 4–5 % DSS in drinking water for 7 days and normal drinking water for 7 days. The arrow indicates CAC. **a**-*right*, Histology of CAC. CAC tissue was fixed and stained with H&E. B-*left*, Expression of IL-6 and SOCS3 mRNA. Total RNA was isolated from colon tissues of chronic colitis or CAC mice. Quantitative RT-PCR was performed using specific primer sets. The data are represented as the mean ± SD (*n* = 10). **b**-*right*, Expression of phosphorylated transcription factors in the mucosa of colitis or CAC mucosa. Colonic tissue homogenates were subjected to Western blotting with polyclonal antibodies against phospho-Stat3, phospho-SHP-2, phospho-Stat1, phospho-NFκB and phospho-38MAPK. C-*left*, Incidence of CAC. During the induction of CAC, sgp130Fc (500 or 50 μg/mouse) or vehicle was injected *i.p.* into BALB/c mice on the first day of each 6–9 DSS cycle (*n* = 10). **c**-*right*, Western blot analysis of phospho-Stat3, phospho-NFκB, TACE, phospho-38MAPK and β-catenin in colonic tissue of sgp130Fc- or vehicle- treated mice. D-*left*, Incidence and number of CAC. During CAC induction, the mice were treated with LcS, PS-PG1-deficient LcS (LC^ΔPS-PG1^) or Saline orally (5 days per week). **d**-*right*, Quantitative RT-PCR analysis of IL-6 and SOCS3 mRNA in colonic tissues in CAC-induced mice treated with LcS, LC^ΔPS-PG1^, or PBS. *;*p* < 0.05, **;*p* < 0.01, a;*p* < 0.05, aa;*p* < 0.01 LcS versus Ct, c;*p* < 0.05, cc;*p* < 0.01 LcS versus LC^ΔP-SPG1^

The mucosal activation of the IL-6/signal transducer and activator of transcription 3 (Stat3) pathway is important for the pathogenesis of IBD and CAC. The inflammatory cytokine IL-6 shows multiple functions and modulates various physiological and immune responses. IL-6 exerts its biological action by binding to two types of membrane receptors, specifically the IL-6 receptor alpha subunit (IL-6Rα) and gp130. IL-6 binds to IL-6Rα at the cell membrane of target cells and this complex in turn associates with gp130, inducing signal transduction via phosphorylation of Stat3. IL-6Rα is expressed on specific cells, such as neutrophils, macrophages, hepatocytes, and several lymphocyte subsets, whereas gp130 is expressed on the cell surface of various cell types. Through this mechanism, the canonical IL-6 signal can transmit their signal to limited cells, which express the IL-6Rα. Suppressor of cytokine signaling 3 (SOCS3) is IL-6/Stat3 responsive protein which inhibits phosphorylation of Stat3 by binding to Janus kinase (JAK) and negatively regulates IL-6-induced signaling. In patients with CD and in murine models of CD (SAMP1/Yit), expression of IL-6 and SOCS3 in the gut is enhanced and Stat3 is excessively phosphorylated [21]. It was also reported that there is an increase in the serum levels of soluble form of IL-6Rα (sIL-6Rα) under inflammatory conditions. sIL-6Rα is produced either by TNFα converting enzyme (TACE), which proteolytically cleaves extracellular domain of membrane-bound IL-6Rα, or by the differential splicing of IL-6Rα mRNA. IL-6 shows an affinity to sIL-6Rs, forming the IL-6/sIL-6Rα complex that can interact with gp130 and induce IL-6 signal transduction, termed IL-6 *trans*-signaling. IL-6 *trans*-signaling can transmit the IL-6 signal in the cells that express only gp130 and promote an inflammatory response through phosphorylation of Stat3. Because the expression of gp130 is ubiquitous, there is a non-specific enhancement of IL-6 *trans*-signaling is thought to be an enhancer of canonical IL-6 signal pathway. The importance of IL-6 *trans*-signaling in the etiology of several chronic inflammations, such as asthma, colitis, and rheumatoid arthritis, has been well documented [22–24]. We previously revealed that the activation of IL-6/Stat3 pathway via IL-6 *trans*-signaling plays a crucial role in the development of ileitis in SAMP1/Yit mice and murine CAC models [25]. We induced CAC in BALB/c mice by administering 9 cycles of treatment with 4–5 % DSS in drinking water for 7 days and normal drinking water for 7 days. CAC was microscopically observed in >60–80 % of mice after DSS treatment. Histologically, we observed the proliferation of gland epithelial cells, resulting in the formation of a polypoid mass (Fig. 2a). Our hypothesis of the association between IL-6 *trans*-signaling and inflammation-based colon tumorigenesis is shown in Fig. 1. IL-6 *trans*-signaling triggered in LP inputs its downstream signal into intestinal epithelial cells (IECs) and induces the expression of anti-apoptotic gene and AID and the production of reactive oxygen species (ROS), which leads to the inhibition of cell death, genetic instability and DNA damage. Mice which are deficient in both IL-10 and AID do not develop colon cancer, whereas IL-10-deficient mice develop spontaneous colon cancers [26]. Aberrant AID expression in the inflamed colonic mucosa plays an integral role during the development of CAC via accumulation of genetic aberrations. Therefore it is speculated that long-term accumulation of IL-6 *trans*-signaling finally leads to colon tumorigenesis. Interestingly, the expression of IL-6, TACE and phospho-Stat3 in CAC mucosa was higher than those in the colitis mucosa (Fig. 2b). Soluble gp130Fc (sgp130Fc) is a dimerized fusion protein of gp130 that competitively suppresses the activation of IL-6 *trans*-signaling by preventing the interaction between gp130 and the IL-6/sIL-6Rα complex. Treatment of DSS-induced CAC model with sgp130Fc suppressed the expression of phospho-Stat3 and the incidence and number of tumors were reduced, compared with vehicle-treated mice (Fig. 2c). Consequently, it was revealed that the activation of IL-6 *trans*-signaling in colonic mucosa was essential for triggering CAC. In our previous study, we indicated that the main source supplying IL-6 in the development of CAC was macrophages and dendritic cells (DCs) located in colonic lamina propria (LP). Moreover, it became clear that a distinct subpopulation of LPDCs was the main sources of sIL-6Rα. LP macrophages, purified from mice with ongoing chronic colitis, actively cleaved sIL-6Rα into the culture supernatant after stimulation with heat-killed commensal bacteria *ex vivo*. However the presence of a TACE inhibitor markedly reduced this cleavage. This result indicates that gut microbiota participated in the production of sIL-6Rα in colonic LP macrophage through TACE activation. Interestingly, the expression of membrane-bound IL-6Rα was markedly decreased in epithelial cells of chronic colitis and CAC. In contrast, the expression of gp130 was significantly increased in epithelial cells of CAC. Therefore epithelial cells of chronic colitis and CAC are thought to be in a state more suitable for the receiving of IL-6 *trans*-signaling than the canonical IL-6 signal pathway.

### Probiotics as prevention for IBD/CAC

Reverting the disturbances of gut microbiota in patients with IBD and CAC, as previously mentioned, should become the new strategy for treatment. Although several clinical trials using probiotics for patients with IBD have been performed in Japan and overseas, the clinical effects are dependent on the probiotic strain and the schedule of probiotic administration. In the trial for the patients with mildly to moderately active UC received one of the probiotic *Lactobacillus* strains, *Lactobacillus casei* strain Shirota (LcS), daily for 8 weeks, significantly better clinical activity index scores were seen after LcS treatment compared with pre-treatment and control group values [27]. In the trial for people at high-risk of developing colorectal cancer, they were administered wheat bran, LcS, both or neither. Incidence of tumors with a grade of moderate or high atypia was significantly lower in the group administered LcS than the other groups. No significant difference in the development of new colorectal tumors was observed with administration of either wheat bran or LcS [28]. After 1 year of treatment with *Bifidobacterium breve* strain Yakult and galacto-oligosaccharides symbiotics, the clinical status was significantly improved, and the amount of myeloperoxidase in the lavage, the number of *Bacteroidaceae* in feces and fecal pH was reduced in the patients with mild to moderate UC [29]. We had reported previously that LcS has the protective efficacy against CAC [30]. LcS suppressed the development of CAC by suppressing IL-6 *trans*-signaling in a murine CAC model, whereas polysaccharide-peptidoglycan complex 1 (PS-PG1) deficient LcS strain had no effect on the prevention of CAC (Fig. 2d). It was also revealed that this effect of LcS was accompanied with improvement of dysbiosis of gut microbiota. As recently reported, transplant of fecal microbiota from healthy individuals is effective in treatment. However transplant of feces requires considered attention in patients with benign disorders, such as IBD, because there is a possibility of accidental contamination with unknown infectious diseases. By avoiding this, probiotics have guaranteed safeguards against such events. Therefore amelioration of dysbiosis by using probiotics could be a potent tool implemented as a new medical treatment for these diseases as well as their prevention.

## Conclusion

Gastrointestinal mucosa has a unique immune system, in which many immune cells reside and exert multiple functions. Those immune cells and gut microbiota have a mutual influence on immune response. Recent studies suggested that an imbalance of gut microbiota—“dysbiosis”—is related to the condition of patients with not only gastrointestinal diseases but also other diseases. It is estimated that dysbiosis of gut microbiota plays an essential role in the initiation of IBD and CAC. The strategy of recent studies on IBD and CAC is altering, which aimed at remedying dysbiosis by considering gut microbiota as “a microbial community effect” from studies focused on individual enterobacterial roles. Dysbiosis of gut microbiota presumably induces the change in the enteric environment that leads to mucosal inflammation or tumorigenesis. It will be necessary to accumulate a scientific overview for the interaction of gut microbiota and the mucosal immune system in the future, to utilize the improvement of the gut microbiota as a mode of treatment in the development of new cures for IBD and CAC.

## Abbreviations

AID: activation induced cytidine deaminase
AOM: azoxymethane
CAC: colitis-associated cancer
CD: Crohn’s disease
DC: dendritic cell
DSS: dextran sulphate sodium
ETBF: enterotoxigenic *Bacteroides fragilis*
GOS: galacto-oligosaccharide
IBD: inflammatory bowel disease
IEC: intestinal epithelial cell
JAK: Janus kinase
LcS: *Lactobacillus casei* strain Shirota
LP: lamina propria
OTU: operational taxonomic units
PD-1: programmed cell death-1
pks: polyketide synthase
PS-PG1: polysaccharide-peptidoglycan complex 1
SOCS3: suppressor of cytokine signaling 3
Stat3: signal transducer and activator of transcription 3
TACE: TNFα converting enzyme
TLR: Toll-like receptor
UC: ulcerative colitis
